# Quantitative norovirus viral load is not affected by home storage of stool

**DOI:** 10.1111/tid.13826

**Published:** 2022-04-01

**Authors:** Michael G. Ison, Ming Tan, Amna Daud, Pengwei Huang, Jason Xi Jiang

**Affiliations:** ^1^ Divisions of Infectious Diseases Northwestern University Feinberg School of Medicine Chicago Illinois USA; ^2^ Divisions of Organ Transplantation Northwestern University Feinberg School of Medicine Chicago Illinois USA; ^3^ Division of Infectious Diseases Cincinnati Children's Hospital Medical Center Cincinnati Ohio USA; ^4^ Department of Pediatrics University of Cincinnati College of Medicine Cincinnati Ohio USA

**Keywords:** frost‐free refrigerator, home storage, norovirus, quantitative viral load, stability

## Abstract

In preparation of a clinical trial of norovirus treatment, there were concerns raised by FDA about risk of self‐storage of stool from patients infected with norovirus affecting quantitative assessments of norovirus RNA. Specifically, most home freezers are frost‐free and may expose the samples to multiple rounds of freeze‐thaw. Stool samples collected by the study team were stored at different lengths in a frost‐free freezer and at −80°C. Quantitative PCRs of norovirus were performed on all samples using the same assay. By all measures, there was no significant change in measured viral load with home storage.

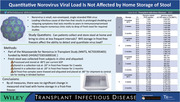

## 1 BACKGROUND

Gastroenteritis is defined as a self‐limiting diarrheal illness that is often accompanied by nausea, vomiting, fever, or abdominal pain. In the United States, norovirus is the single most common cause of acute gastroenteritis that leads to medical evaluation in adults, and the second most common cause of severe diarrhea in infants and young children.^1^, ^2^ Increasingly, norovirus is recognized as a common cause of chronic gastroenteritis in immunocompromised patients. Prolonged diarrhea can lead to dehydration, allograft dysfunction, renal insufficiency, and rarely death in these patients.^3^, ^4^ Treatment is generally supportive, although a range of agents, including immunoglobulin, breast milk, and nitazoxanide have been used clinically.^2^

Noroviruses are small, nonenveloped, single‐stranded ribonucleic acid (RNA) viruses that are members of the *Caliciviridae*.^1^ Norovirus is highly contagious as transmission is highly efficient with a median infectious dose of 18 viruses. There are generally high titers of virus within stool (10^5^ to 10^10^ genome copies per gram of feces); therefore contact with infected feces and vomit, fecally‐contaminated food, water, and surfaces are common sources of infection.^5^ Norovirus is also highly stable with retained infectivity under environmental conditions that would generally inactivate other viruses, including chlorine at concentrations used in drinking water.

The virus is viable under a wide range of conditions for a prolonged period of time. Studies to assess stability of virus have generally utilized the cultivable murine norovirus, Tulane virus, and feline calicivirus to predict the infectivity of human norovirus.^6^, ^7^, ^8^ Such surrogate‐based studies have estimated that human norovirus could stay potentially infectious on frozen foods (less than or equal to −20°C) and refrigerated foods (≤10°C) for up to 6 months and up to 7 days.^5^, ^6^, ^7^

While conducting a clinical trial of nitazoxanide against norovirus in transplant recipients (NNITS, ClinicalTrials.gov Identifier: NCT03395405), enrollment was challenged, in part, by patients need to frequently present to clinic to provide stool specimens for quantitative polymerase chain reaction (PCR) of norovirus, which was a key secondary outcome measure of the study. It was felt one way to reduce the need for visits would be to have patients collect and store stool using standard methods and containers at home. In reviewing our proposal, the Food and Drug Administration (FDA) noted that viral RNA in any clinical matrix, but particularly in stool, is not stable, especially if it is subjected to multiple rounds of freeze‐thaw that could result in the breakdown of viral particles. Further, home self‐storage would require use of self‐defrosting home freezers, which would further expose the samples to multiple rounds of freeze‐thaw and compromise the integrity of the viral RNA samples.

Given the known stability of virus in traditional freezers and to address the concerns raised by the FDA, we utilized residual samples collected and processed in our existing protocol and studied quantitative viral measurements after freezing at −80° and after freezing in a frost‐free freezer for 2 weeks and 2 months.

## 2 METHODS

Samples for this study were conducted as part of our IRB‐approved protocol for the nitazoxanide against norovirus in transplant recipients study (NNITS, ClinicalTrials.gov Identifier: NCT03395405); retention and use of residual stool, which is defined as stool retained beyond what is required for study‐specified assays for future use, was permitted per protocol. This protocol, which was conducted under IND 139,710, was approved to proceed by FDA. Only samples from patients collected at Northwestern University (NU) were utilized for this sub‐study. Patients had to collect a sample of stool, ideally on the morning of the study visit; the sample was to be stored in a provided container in two plastic bags in a standard refrigerator for no more than 24 h prior to sample preparation in the laboratory. One gram aliquots of the stool were prepared. Three aliquots were either placed immediately in a −80°C freezer for this study, and additional aliquots were stored per protocol for primary study procedures; the stool was divided into two approximately equal parts and placed in a standard plastic container used for home collection and storage. This was double bagged per our patient instructions and then placed in a frost‐free freezer (Euhomy 1.1 cubic foot mini freezer) and stored there for 2 weeks or 2 months. The stool remained frozen the entire time that it was in storage. After 2 weeks, one tub was allowed to sit at room temperature for 1 h and then taken to the laboratory where it was further aliquoted into four 1‐gm aliquots (one for retention at NU and the others to be sent to the Central Lab at Cincinnati Children's Hospital Medical Center). These were all frozen at −80° until all samples were ready to be sent to the Central Lab. After 2 months, the last tub was handled as above. Samples were labeled with a random set of numbers with a file maintained under password protection at NU so that the Central Lab would not know which samples were from which patient or storage condition. Samples were sent to the Central Lab with sufficient dry ice to ensure that samples remained frozen for the entire duration of shipping. Upon arrival, the Central Lab confirmed that samples were frozen, and there was residual dry ice when the box was opened after arrival in the lab. This key was never shared with the Central Lab. When all relevant samples were collected and processed, they were sent to the Central Lab.

Samples were sent to the Central Lab where they were processed to determine norovirus loads using standard quantitative PCRs (qPCRs).^9^ To this end, two pairs of degenerated primers, each being able to amplify GI (CGCTGGATGC GNTTCCAT [named QNIF4]/CCTT AGACGCCATCATCATTTAC [named NV1LCR]) or GII (ATGTTCAGRTGGATGAGRTTCTCWGA [named QNIF2D] /TCGACGCCATCTTC ATTCACA [named COG2R]) noroviruses, as well as two probes that are specific to GI (6FAM‐TGGACAGGAGAYCGCRATCT‐MGBNFQ, named NV1LCpr) and GII (6FAM‐TGGGAGGGCGAT CGCAATCT‐MGBNFQ, named RING2‐TP) amplicons, respectively, were designed based on the conserved genomic regions encoding the RNA‐dependent RNA polymerase (RdRp). The primers (Integrated DNA Technologies) and the fluorescently labeled probes (Thermo Fisher Scientific) were synthesized using commercial services. GI and GII norovirus RNAs that were isolated from our standard norovirus positive stool pools were used as standard RNAs to make standard curves. The norovirus RNAs were quantitated using commercial norovirus viral RNAs (American Type Culture Collection [ATCC]) with known concentration (genome copies/μl). The stool samples were first diluted 1:10 in nuclease‐free water for noroviral RNA extraction as described previously.^10^ The RNAs were then used for reverse transcription qPCRs using TaqMan Fast Virus 1‐Step Master Mix (ThermoFisher Scientific) approach. The used thermal cycle included 50°C for 10 min for reverse transcription; 95°C for 10 min for cDNA denaturation, followed by 45 cycles of 95°C for 15 s and 60°C for 45 s, which ended with an additional step of 60°C for 1 min as final extension. The reaction volume for the PCR was 20 μl. The outcomes in Ct (cycle threshold) values were calculated to norovirus loads in genome copies/ml 10% stool using the standard curve (see above) with an efficiency of 96.5%. PCR controls included (1) a negative RNA extraction control from the RNA extraction step using known stool without noroviruses; (2) a positive RNA extraction control from the RNA extraction step using known stool containing noroviruses; and (3) a PCR negative control using nuclease‐free water to replace RNA extraction. Raw results were shared with the NU team that linked the sample ID. The NU team then used the key to assess, which aliquots were from each patient and storage condition.

## 3 RESULTS

Stool samples were collected from two subjects at five different study visits (Subject 02ENW004 on visit 4; Subject 02ENW005 on visits 1, 3, and 8). The norovirus loads in the samples were determined by qPCRs that were run in triplicates for each specimen. Only GII noroviruses were detected, and the results are shown for each sample and storage condition in Table 1. For each of the four samples, there is far less than 0.5 log difference in the genome copies/ml 10% stool across the three storage conditions (i.e., 6.2 log_10_ copies/ml in all three conditions for sample #1). In no sample was there a reduction in the viral titer comparing samples stored at −80° or stored in a frost‐free freezer for up to 2 months.

**TABLE 1:** GII noroviral loads in Ct, genome copies/PCR reaction, and genome copies/ml 10% stool of stool stored at −80°C or in a frost‐free freezer for indicated times

Sample	Storage	Cт	Genome copies/PCR reaction	genome copies/ml 10% stool (x 214.28571)
1	Immediate −80°C	22.4	7202	1 543 261
		22.3	7514	1 610 157
		22.5	6798	1 456 688
	2 weeks	22.5	6605	1 415 360
		22.6	6514	1 395 907
		22.5	6862	1 470 385
	2 months	22.4	7060	1 512 757
		22.2	8094	1 734 332
		22.4	7109	1 523 305
2	Immediate −80°C	22.4	7480	1 602 788
		22.3	7689	1 647 722
		22.4	7181	1 538 945
	2 weeks	21.9	10412	2 231 192
		21.8	10 546	2 259 763
		22.0	9713	2 081 422
	2 months	22.5	6644	1 423 812
		22.4	7265	1 556 732
		22.5	6943	1 487 714
3	Immediate −80°C	24.9	3115	667 571
		24.9	3010	645 104
		24.9	3068	657 418
	2 weeks	23.5	3393	727 074
		23.6	3196	684 951
		23.7	3096	663 485
	2 months	23.0	4769	1 021 974
		22.9	5238	1 122 397
		23.0	4853	1 039 909
4	Immediate −80°C	20.7	22 376	4 794 805
		20.4	27 487	5 890 081
		20.4	28 046	6 009 957
	2 weeks	21.2	16 142	3 459 005
		21.5	13 568	2 907 438
		21.4	14 494	3 105 915
	2 months	19.8	40 844	8 752 493
		19.9	37 996	8 142 038
		19.9	39 068	8 371 735

*Note*: The multiplier 214.28571 is a factor to convert the value of genome copies/PCR reaction in the genome copies/ml 10% stool that is required by the MOP of the study. This multiplier was calculated based on number 6.022 × 10^2^ ^3^, Avogadro's constant.

## 4 DISCUSSION

These data suggest that there is not significant loss of viral load with the storage of stool in frost‐free freezers. In fact, the variability of the data over time in storage is within 0.5 logs, which is the expected range of values in replicate. Further, there was no decline in detectable virus with longer storage in a frost‐free freezer. As such, these data support the ability for patients to collect and store samples at home and reduce the number of in person visits to potentially improve enrollment of the study. Importantly, such an approach should not jeopardize virologic endpoints and may inform design of future studies.

In our current study, we proposed to have subjects present to clinic for samples to be collected at week 0, 1, 2, 3, 4, 8, 12, and 24 weeks. This represents significant burden on subjects to travel to clinic, mostly to deliver samples to the sites. Most other clinical data can be collected by a verbal discussion with the patient, which could be done at a distance for most appointments. Only visits 0, 1, 4, and 24 require in person visits to also collect blood. In designing the study, we selected 2 week and 2 month storage in a frost‐free freezer as a surrogate of short and longer‐term storage at home with the goal of collecting data quickly to address FDA concerns. If there had been any evidence of loss of agreement between samples stored at −80°C and 2 week or 2 month storage, additional timepoints would have been studied.

Given the lack of significant change in viral load under various storage conditions in addition to prior studies in nonclinical settings demonstrating stability of norovirus for up to 6 months, we did not do additional studies to assess stability beyond 2 months.^5^, ^6^, ^7^ We did note an increase in genome copies after 2 months of storage in samples 3 and 4. It is felt that this increase is small and likely the result of experimental variation.

One limitation is that this study did not assess levels of infectious virus as molecular diagnostics are generally used for most studies of norovirus, and norovirus does not grow in conventional culture systems. Another limitation is that we only had samples of GII norovirus from our subjects. While we did not test other genotypes, it is unlikely that stability would be markedly different across various genotypes. Lastly, we utilized a small sample of stool that was selected for convenience.

These data support the ability to reduce patient burden in clinical trials while maintaining integrity of the key virologic data for norovirus studies. We do acknowledge that the proposed self‐storage of samples may not follow all components of good laboratory practice (GLP). That said, this can mostly be addressed by utilizing standard procedures for collecting and storing of specimens, including using the same storage containers for all subjects. Additionally, having the subject record the details of sample collect, timing of freezing and removal from freezer would allow collecting most critical data. Continuous monitoring of personal freezers is generally not feasible. That said, given that personal freezers are utilized regularly, patients will know if their freezer fails, and, if this occurs, the patients can be directed to call the study team to attempt to bring specimens to the laboratory before they completely thaw and within 24 h of failure. By keeping in good faith with GLP, the integrity of the study can be maintained. Surveys of subjects found broad enthusiasm for self‐storage if it would avoid in person visits.

A key challenge to conducting clinical studies is the frequency of in person visits. Approaches to minimize in person visits should improve patient enrollment and retention. We have demonstrated that self‐storage of stool specimens for quantitative norovirus PCR in frost‐free freezers does not result in change in detection of viral load with storage. The data suggest that such a patient‐focused approach will not jeopardize virologic endpoints and may improve enrollment. As such, it should be considered in future study designs of trials of norovirus epidemiology, prevention, or treatment.

## CONFLICT OF INTEREST

MGI reports research support, paid to Northwestern University, from AiCuris, GlaxoSmithKline, Janssen and Shire; he is a paid consultant for Adagio, AlloVir, Celltrion, Cidara, Genentech, Roche, Janssen, Shionogi, Viracor Eurofins; he is also a paid member of DSMBs from Janssen, Merck, SAB Biotherapeutics, Sequiris, Takeda and Vitaeris; he also receives royalties from UpToDate.

MT, AD, PH and JXJ have no potential conflict of interest to declare.

## AUTHOR CONTRIBUTIONS

MGI obtained funding for the study, conducted the study, and wrote and revised the paper. MT, PH, and JXJ conducted the study and wrote and revised the paper. AD revised the paper. All authors approved the final version of the paper.

## Supporting information

Visual Abstract
