# Supplementary material for: Quantitative norovirus viral load is not affected by home storage of stool
Source: Transpl Infect Dis. 2022 Apr 1;24(3):e13826. doi: 10.1111/tid.13826 (PMC9233081; doi:10.1111/tid.13826)
Supplement: Supplementary file 1 — Visual Abstract [file TID-24-e13826-s001.pptx]

## Slide 1
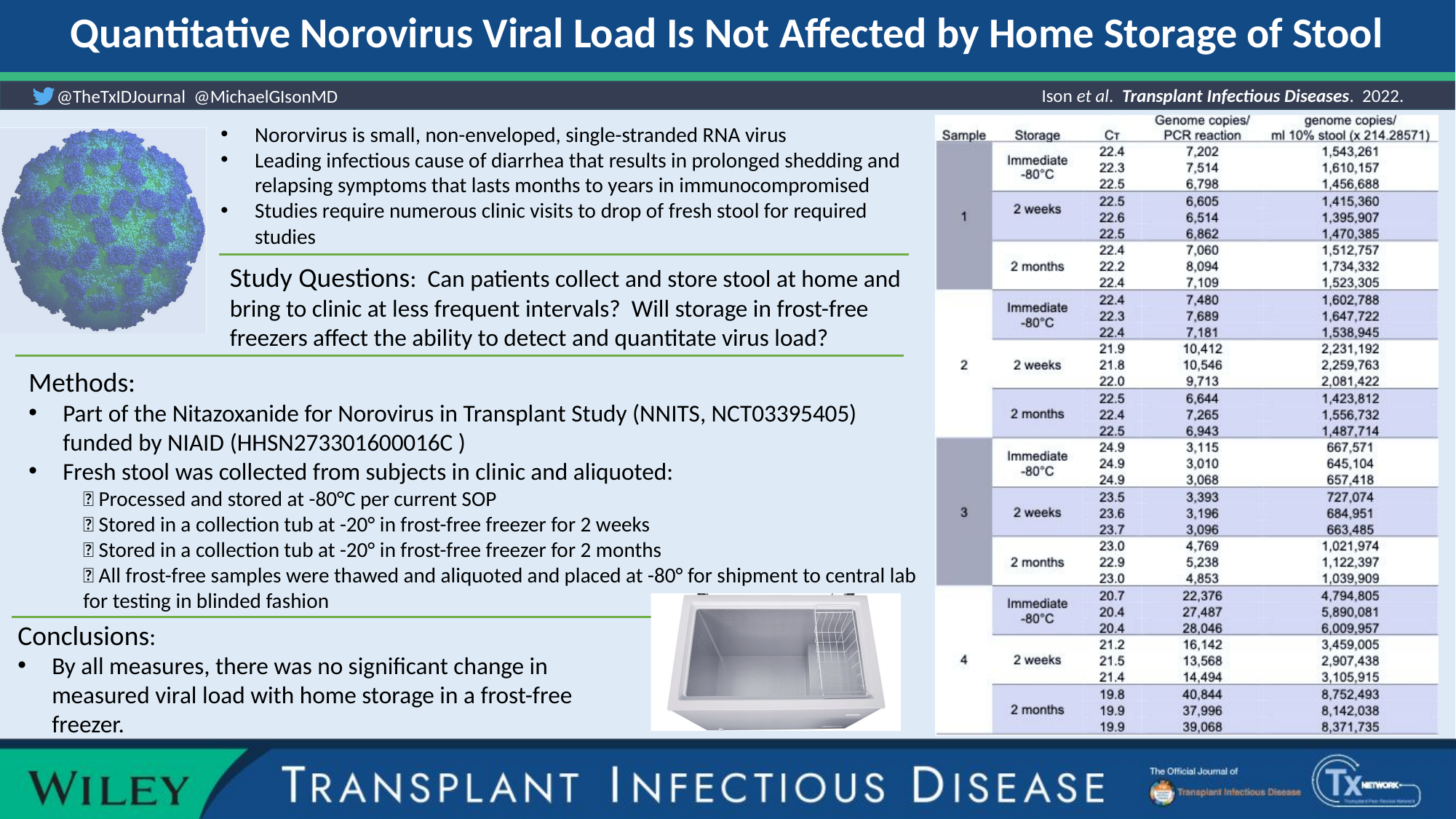

Quantitative Norovirus Viral Load Is Not Affected by Home Storage of Stool
Ison et al. Transplant Infectious Diseases. 2022.
 @TheTxIDJournal @MichaelGIsonMD
Nororvirus is small, non-enveloped, single-stranded RNA virus
Leading infectious cause of diarrhea that results in prolonged shedding and relapsing symptoms that lasts months to years in immunocompromised
Studies require numerous clinic visits to drop of fresh stool for required studies
Study Questions: Can patients collect and store stool at home and bring to clinic at less frequent intervals? Will storage in frost-free freezers affect the ability to detect and quantitate virus load?
Methods:
Part of the Nitazoxanide for Norovirus in Transplant Study (NNITS, NCT03395405) funded by NIAID (HHSN273301600016C )
Fresh stool was collected from subjects in clinic and aliquoted:
💩 Processed and stored at -80°C per current SOP
💩 Stored in a collection tub at -20° in frost-free freezer for 2 weeks
💩 Stored in a collection tub at -20° in frost-free freezer for 2 months
💩 All frost-free samples were thawed and aliquoted and placed at -80° for shipment to central lab for testing in blinded fashion
Conclusions:
By all measures, there was no significant change in measured viral load with home storage in a frost-free freezer.
